# Explaining the Sentence Superiority Effect and N400s Elicited by Words and Short Sentences with OB1-Reader

**DOI:** 10.5334/joc.358

**Published:** 2024-04-17

**Authors:** Noor Seijdel, Gina Stolwijk, Beatriz Janicas, Joshua Snell, Martijn Meeter

**Affiliations:** 1 Department of Educational and Family studies, Vrije Universiteit Amsterdam, Amsterdam, the Netherlands; 2 LEARN! Research Institute, Vrije Universiteit Amsterdam, the Netherlands; 3 Department of Experimental and Applied Psychology, Vrije Universiteit Amsterdam, Amsterdam, the Netherlands; 4 Institute of Brain and Behavior Amsterdam (iBBA), Amsterdam, the Netherlands

**Keywords:** Reading, word recognition, computational modeling, EEG, N400

## Abstract

Research into reading has benefitted from the emergence of powerful computational models that account for reading behavior at different levels. Such models become more powerful when the underlying anatomy, architecture or ‘physiology’ can be linked to the behavior of interest. OB1-reader is a reading model that simulates the processes underlying reading in the human brain. Previous studies showed that OB1-reader can account for various phenomena in the word recognition and text reading literatures. Here we aim to extend OB1’s scope, by simulating behavioral performance and evoked EEG activity for two experimental word-recognition tasks: a flanker task in which unrelated flankers generated less accurate responses combined with a larger N400, and a sentence reading task in which words were recognized more accurately at central positions and within intact sentences, than at peripheral positions and in scrambled sentences. OB1 simulated several behavioral findings in both paradigms, including the so-called sentence superiority effect. Moreover, virtual event-related potentials (ERPs) generated from node activity in OB1 were compared to human ERPs. More lexical activity in OB1 predicted the size of the N400 component of human readers in both experiments, but not the N250.

## Introduction

One way to understand how the human brain processes information involves building computational models that account for behavior under different conditions. Such models become more powerful when the underlying brain anatomy and physiology can be linked to the behavior of interest (Meeter, Jehee, & Murre, 2007).

A field in which computational modelling has had a large impact is research into reading. Reading is a complex process, in which the observer engages with a text at multiple levels simultaneously, from recognition of single words, to moving through a sentence, to comprehending the meaning of the text. In each of these steps, elaborate computational models have inspired ever-cleverer experiments to test their predictions. In the literature on word recognition, for example, the need to adjudicate between several successful computational models (e.g., Davis, 2010; Gomez, Ratcliff, & Perea, 2008; Grainger & van Heuven, 2003) has allowed researchers to explain subtle effects related to orthographic processing, such as effects of letter transpositions (see Grainger, 2008 for a review). In the literature on text reading, the success of models based on serial processing of words (most prominently, E–Z Reader, Reichle et al., 1998; Reichle, Warren, & McConnell, 2009), as well as models based on parallel processing of words (most prominently, SWIFT, Engbert et al., 2005; Rabe et al., 2021), has led to creative studies of how words influence one another while a reader goes through a text (e.g., Angele, Tran, & Rayner, 2013; Dare & Shillcock, 2013; Snell, Meeter, & Grainger, 2017; Snell, Vitu, & Grainger, 2017). Recently, these two strains of computational models - of word recognition and of text reading- were combined into one overarching model, the Open Bigram Reader 1 or OB1-Reader (short: OB1, Meeter et al., 2020; Snell et al., 2018).

While OB1 and other computational models have been successful at explaining findings at the behavioral level, none so far has been linked convincingly either to brain structures, or to processing in the brain. Crucially, in explaining behavioral phenomena different models have frequently been shown to do an equally good job (e.g., SWIFT and E–Z Reader are on par in explaining eye movements). Investigating how well models may account more directly for brain activity offers a potential new means to adjudicate among theories. As a proof-of-concept, here we investigate whether OB1 can simultaneously predict behavioral and electrophysiological responses elicited in reading and word recognition tasks.

### Event-related responses elicited by reading

While fMRI studies of reading are growing apace (e.g., Murphy, Jogia, & Talcott, 2019), the technique that has been used most to investigate reading is electrophysiology (EEG). With this technique electrical activity in the brain is measured through electrodes on the scalp. On any one trial this activity cannot be separated from background noise, so data from multiple trials with the same stimulus or situation is averaged to yield an *evoked response potential* or ERP.

Reading words evokes reliable ERPs, consisting of a cascade of ERP components thought to reflect the different processes involved in visual word recognition (Grainger & Holcomb, 2009; Holcomb & Grainger, 2006). These include the mapping of visual features onto letter representations (N/P 150), the processing of sublexical orthographic and phonological information (N250), the processing of word identities and mapping these onto semantic information (N400). Here we focus on the latter two components.

The N250 is a negative-going component that peaks around 250 ms after presentation of words (Grainger, Kiyonaga, & Holcomb, 2006; Holcomb & Grainger, 2006). It is the earliest component that is sensitive to the lexical status of a word (Duñabeitia et al., 2009), suggesting that it might be indexing the integration of orthographic information to lexical representations.

The N400, a negative deflection of the ERP at around 400 ms after word presentation (Kutas & Hillyard, 1980), is traditionally seen as an index of semantic processing – it is, e.g. larger for words that do not fit the context and might thus require more processing (Bentin, Kutas, & Hillyard, 1995). More recently, it is often interpreted as indexing that predictions have been violated (Kuperberg, Brothers, & Wlotko, 2020; Lau, Holcomb, & Kuperberg, 2013; Levy, 2008; Nour Eddine, Brothers, & Kuperberg, 2022) – that, for example, an article is being processed that is incompatible with an expected noun (DeLong, Urbach, & Kutas, 2005) – although a well-powered replication study could not find this particular effect (Nieuwland et al., 2018). While several pieces of evidence indeed point to a role for violated predictions (DeLong & Kutas, 2020; Kuperberg, Brothers, & Wlotko, 2020; Lau, Holcomb, & Kuperberg, 2013), other empirical results are more naturally explained by a hypothesis in terms of the amount of lexical activation. For example, the observation that semantic priming reduces the N400 was first taken to indicate that overlapping semantic features would reduce processing of the primed word, leading to a lower N400 (Holcomb, 1988; Lau, Holcomb, & Kuperberg, 2013). Moreover, an unpredictable word that is semantically related to context words produces a smaller N400 than a similarly unpredicted word not related to the context (DeLong & Kutas, 2020) This again suggests that the N400 is sensitive to whether a word activates semantic features not yet activated by the context.

Even more basically, some studies have suggested that the N400 is also sensitive to the number of activated lexical units. For example, words generate a larger N400 when they have more orthographic neighbors (Holcomb, Grainger, & O’rourke, 2002; Laszlo & Federmeier, 2009), presumably because when the word is presented those neighbors (e.g., word-work) are also activated. More directly, Snell et al. (2019) compared ERPs generated by either a target word presented alone, presented with two unrelated flanking words, or the target word repeated three times to control for the size of the stimulus. When either target words alone or repeated targets were responded to faster, the N400 was largest in the condition with different flankers, in which multiple stimulus words could activate their associated lexical representations. Of course, these lexical representations may in turn activate semantic features that may be the true driver of the N400. Irrespective of this second step, the sensitivity of the N400 to how many lexical items are activated allows this component to be used as a measure of lexicon activation, which is otherwise unobservable. Here, we use this characteristic to test the plausibility of OB1-reader.

### Approach

Previous research has already shown that computational models can simulate N400 activity. Models like those developed by Cheyette and Plaut (2017), Laszlo and Plaut (2012), and Rabovsky and McRae (2014) have successfully simulated N400 effects in various contexts, such as word repetition and semantic congruence in sentences. However, these models often do not capture the dynamic nature of the N400’s rise and fall and, more importantly, lack the ability to simulate behavioral responses alongside neural activity (Nour Eddine, Brothers, & Kuperberg, 2022). Our approach is a different one. We test whether OB1, without altering to its structure or parameter values, can simulate behavioral performance and the evoked N400 activity simultaneously. We generate virtual event-related potentials (ERPs) by simply summing the lexical activity generated by OB1 in the N400 time window. This aligns with the putative biological generation of EEG (Murakami & Okada, 2006). This study, therefore, seeks to fill a critical gap by not only providing a link between computational predictions and brain activity but also by incorporating both behavioral and electrophysiological data in the modeling of reading processes. This approach represents a novel and significant advancement in understanding the neural correlates of reading and word recognition.

We compare OB1’s virtual ERPs to human ERPs in two ways: first we compare conditions as they have been defined in the experiments. Snell et al. (2019) compared ERPs generated in three flanker conditions. We simulate these by presenting the exact same stimuli, using the experiment’s timing, to OB1. Then, we look at the virtual ERP predicted by OB1 for individual stimuli, and test whether these virtual ERPs correlate with the ones observed in the experiment. Since this involves regrouping stimuli in a way that was not originally published, this is a blind test of OB1’s ability to predict ERPs, and, consequentially, the model’s plausibility.

While OB1 was tested exactly as it was in our simulation of Snell et al. (2019), a second experiment for which stimuli and EEG results were available to us required the introduction of another component to OB1. Wen et al. (2019) shortly presented four-word sentences to participants, either in their regular, grammatical order or a scrambled order, while EEG was recorded. Participants then had to report one of the words, indicated by a probe dot that appeared only after the sentences. Their main findings were that participants could recognize words better when they were in a grammatical sentence, the so-called sentence superiority effect. In OB1 as published by Snell et al. (2018), semantic predictions were assumed to aid in recognizing words. Here, we extend that principle to syntactic categories: we allowed OB1 to predict the syntactic categories of unrecognized words surrounding a recognized word. We then tested whether with this mechanism OB1 could reproduce the sentence superiority effect, and whether it would generate plausible ERPs for Wen et al.’s simple sentences. This is a computational implementation of the syntactic feedback assumption as described in, among others, Snell et al. (2017) and Snell and Grainger (2019).

## Methods

### The OB1-reader model

**Figure 1:**
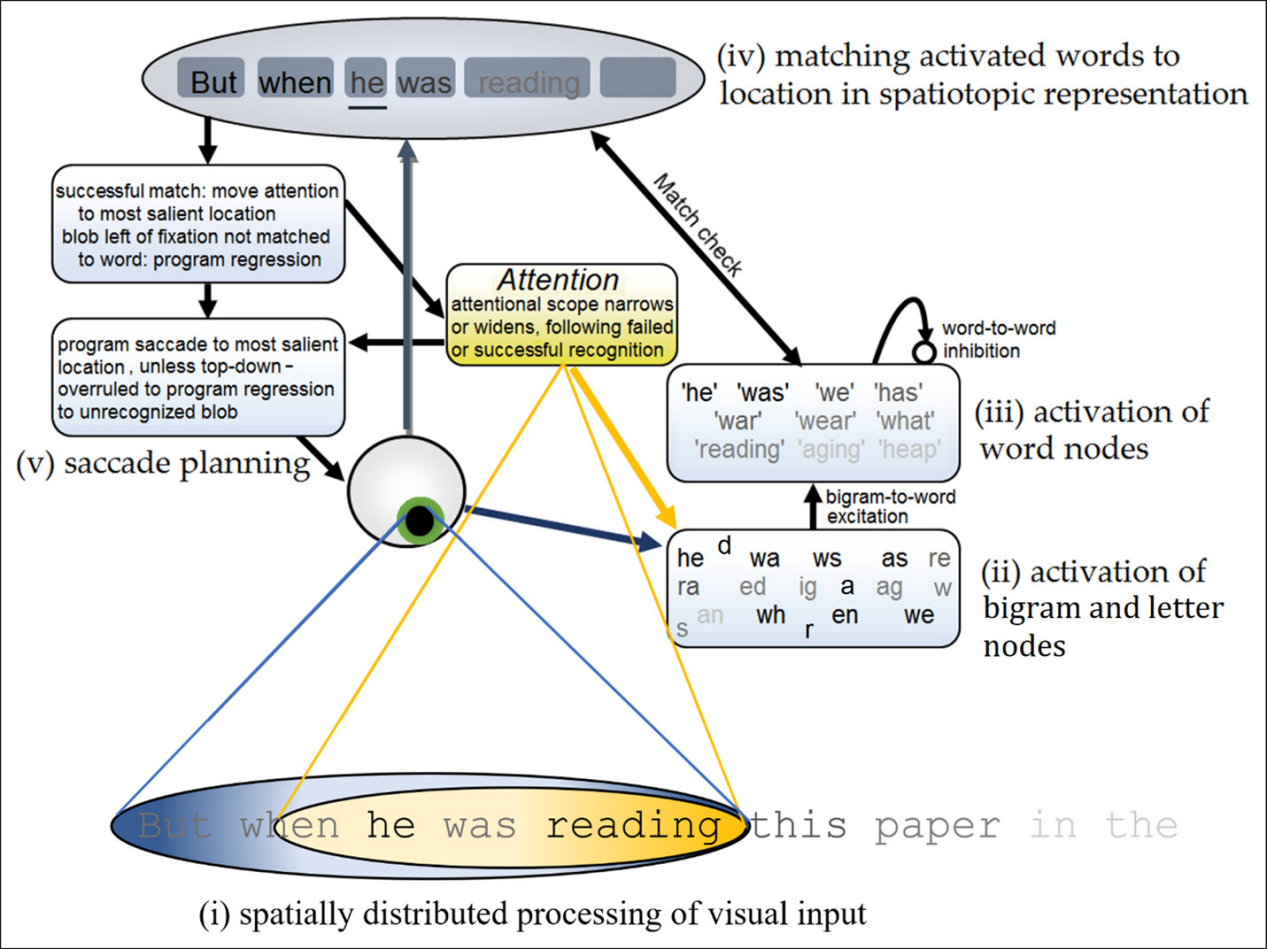
**Schematic diagram of OB1-reader. (i)** OB1 sees multiple words at a time. Within the visual input, letter processing is modulated by visual acuity (shown through the letter contrast) and visuo-spatial attention (yellow oval). The focus of attention can shift relative to the eye’s fixation. **(ii)** Open-bigram nodes are activated by the visual input, with stronger activation of letters that suffer less from crowding because they are at a word edge, and that are close to the centers of fixation and attention. **(iii)** Word nodes are activated by bigram nodes coding for the open bigrams that occur in the word (e.g., ‘reading’ by ‘re’ but also ‘ra’). Word nodes are inhibited by word nodes that share the same bigrams. **(iv)** Upon fixating a text, OB1 generates a spatiotopic sentence representation that codes the location and approximate length of yet-to-be-identified individual words. Word nodes that reach a recognition threshold are matched to locations (‘blobs’) in the spatiotopic representation based on length. OB1 only recognizes a word when it can be mapped onto a plausible location. When a word is successfully recognized, attention moves ahead of the eyes to the most salient word (defined by its number of letters weighed by their proximity to the centers of fixation and attention). **(v)** Whether a saccade is initiated is stochastically determined in each 25 ms processing cycle, with successful word recognition increasing the chance of initiation. The center of the most salient word becomes the saccade target. Saliency-based saccade targeting is overruled when a word location to the left of fixation has not yet been marked as recognized. Instead, a regression to that location will be executed. The attentional window is widened after each fixation during which a word is successfully recognized, while it is narrowed after each fixation without successful recognition.

OB1 Reader consists of five parts (see Figure 1) that will be introduced below (details can be found in Meeter et al., 2020; Snell et al., 2018).

Visual input to the model is a line of text centered around fixation, with visual acuity and visual attention limiting what information is picked up from this input. To account for crowding effects letters in the centers of words receive weaker visual input than letters at the edges of words (see Grainger, Dufau, & Ziegler, 2016 for a discussion of the combined effects of crowding and acuity; Grainger, Tydgat, & Isselé, 2010; Perea & Gomez, 2012).

Visual input is assumed to activate letter units and so-called open bigram units, that code for the presence of two letters within a letter string in the input in a certain order, though not necessarily in adjacent positions (Grainger & van Heuven, 2003). For example, presentation of the word ‘READ THIS’ activates the bigram unit for ‘RE’ and ‘HI’, but also for ‘RD’ and ‘HS’ (Note that ‘RT’ would not be activated because ‘R’ and ‘T’ are in different letter strings). Letter and bigram units receive input from the whole input array; they are location-invariant.

Letter and bigram units in turn activate units coding for words. Activity of word units is updated in discrete 25 ms time steps according to standard interactive activation formulas (McClelland & Rumelhart, 1988). Word units receive excitation from units coding for letters and open bigrams contained within the word (e.g., the unit for ‘READING’ is activated by letter- and bigram units R, A, RE, RD, DN, etc.). Word units receive inhibition from other word units, scaled by the number of letters and bigrams that these words share. The assumption underlying this is that inhibitory connections are only formed between words that are often active at the same time, because they share the same inputs. This inhibition helps the model avoid activating multiple words that are plausible interpretations of the same letter string (e.g. “this” and “his” when the input is THIS), but does not hinder the activation of word units coding for non-overlapping words (e.g., “read” and “this” when the input is READ THIS).

Visual input also sets up a spatiotopic representation of how many words there are, and what their approximate length is. This representation initially has the form of unidentified blobs, but as word nodes are activated, a matching process assigns word identities to blobs that match in length.

The model contains a system to move an attentional window and plan saccades. In reading, the spotlight moves to the visually most salient location (determined by word size, eccentricity and closeness to the focus of attention) to the right of the current focus of attention, followed by a saccade to the location of the attentional window. However, when the spatiotopic representation contains an unrecognized word to the left of fixation, the spotlight moves to the location of that unrecognized word, and a regression is planned. In the two paradigms simulated here saccades played no role, since stimuli were presented too short to allow participants to make saccades within it. Instead, participants were assumed to fixate in the center and set their attentional window broad enough to capture the whole stimulus.

To the number of 25 ms time steps that it takes the model to recognize a word, a fixed sum was added of 400 ms for visual processing, decision and motor time. The resulting sum was used as the simulated RT in, for example, lexical decision tasks.

Since OB1 did not yet “speak” French, a lexicon was created consisting of the 2000 most frequent words in French, in addition to the words present in the experiments. Word frequencies were obtained from the French Lexicon Project (Ferrand et al., 2010).

### Syntactic predictions

For the flanker tasks, OB1 as previously published was used. For the sentence task, however, a new form of prediction was implemented. OB1 assumes that readers continuously predict the next word they are going to read, aiding recognition when the next word matches those predictions. This was modeled using so-called Cloze norms. These are derived from the Cloze task, where readers read a sentence up to a word that is omitted, and have to guess what that word might be. Readers may rely on semantics to make those guesses, but they may also rely on syntax.

For the tasks we simulate here no Cloze norms are available, so we modeled predictions made by readers only from syntactic sources of information. In particular, we assumed that readers can use the probabilities with which one part of speech follows one another to predict the part of speech of not yet recognized words. To make it concrete, a sentence like “She walked to the…” may allow too many continuations to lead to strong predictions for the next word. However, the part of speech of the next word can be confidently predicted to be either a noun or an adjective, since these follow a determiner with some 90% likelihood in English. We assumed that readers would, when they recognize a word in a sentence, use learned likelihoods of transitions between part-of-speech categories, to boost the activation of words with likely part of speech.

The stimuli of Wen et al. were French. Therefore we tabulated the transition probabilities between all Part of Speech categories (i.e., verb-noun transitions, noun-adjective, etc.). These were computed from the French TCOF corpus (Benzitoun, Fort, & Sagot, 2012). Concretely, at the time step that word *n* was recognized in a sentence, the activation of plausible candidates for position *n + 1* (provided that this word was not yet recognized) was increased by adding, for this one time step, c_PoS_ * p(PoS transition) to the activation of the candidate word. Constant *c_PoS_* was set to 0.5, while *p(PoS transition)* was the likelihood of a transition from word *n*’s part of speech to that of word *n + 1* (e.g., from a determiner to a noun).

Crucially, this was done for both the forward transition, as well as the backward transition from word *n*‘s part of speech to that of word *n–1* in case word *n–1* was not yet recognized. This can be considered rare in normal left-to-right reading, but it could occur in Wen et al.’s (2019) sentence reading task that we simulated since participants had to fixate in the center of a sentence and have to recognize words to the left and right of it. For example, if a participant was presented with the sentence “The wolves were hungry”, and recognized “wolves” after two time steps, plausible candidates for the first position (those that had the right length, i.e. three letters, and had above 0 activation after the previous time step) received in this one time step an extra activation equal to c_PoS_ * p(PoS transition). For all active three-word determiners this was an extra 0.5 * 0.55 of activation (since the likelihood of a determiner preceding a noun is 0.55 in French). As another example, for all active three-word adverbs it would be 0.5 * 0.015 extra activation, since adverbs are not likely to occur before nouns in French. More details can be found in Janicas (2022).

### Stimuli and procedure

The behavioral and EEG data used in the current study have been previously described and analyzed to address different questions. A brief overview will be given here; see Snell et al. (2019) and Wen et al. (2019) for further details.

#### Flanker lexical decision task

First, we simulated performance and human EEG activity evoked by targets and flankers during a lexical decision task. We used the experimental stimuli from Snell et al. (2019), consisting of 80 four-letter word targets and 80 four-letter pseudoword targets from the French Lexicon Project database (Ferrand et al., 2010). Stimuli contained no diacritics. For each target, an orthographically unrelated stimulus with the same length, frequency, and lexical status was selected from the database and used as unrelated flankers. The stimulus set contained 240 word and 240 pseudoword stimuli that were counterbalanced across conditions.

During the experiment, foveal target words were presented for 150 ms, and then replaced with a fixation cross until participants responded (with a maximum of 2000 ms). Targets could be flanked on each side by parafoveal words: repetition flankers (e.g. ROCK ROCK ROCK), or unrelated flankers (e.g., STEP ROCK STEP). There was also a no-flanker condition where just the target word appeared (e.g., ROCK). In the simulations, we again used the same stimuli and timing as in the experiments. Pseudoword trials were not simulated, as pseudoword stimuli were merely used to induce the task and were not analyzed by the original authors. For the simulated ERPs, again the activity of all lexical units was summed on each time step.

#### Sentence task

Then, we simulated performance and human EEG activity evoked by four-word French sentences presented either in their normal, syntactically correct order or in a scrambled order. A correct sentence would be “ces loups vont voir” (those wolves will see), while its scrambled version was “ces vont loups voir” (those will wolves see). Wen et al. (2019) showed participants the four-word sentences (either normal or scrambled) for 200 ms, followed by a 500 ms mask and then a post-cue that indicated which word participants had to present. In our simulation of this experiment, we used the stimuli from Wen et al., consisting of 200 sequences of four words with the average word length of 4.03 letters, SD = 0.82. In each sentence one word was designated as the target, with for each position 50 target words. To construct a scrambled version of every sequence, Wen et al. switched the positions of all words except for the target. The same sentences were used in simulations with OB1. We presented each sentence for 200 ms to the model, after which they were taken away. The simulation continued for another 400 milliseconds to allow OB1 to finish word recognition (the task of Wen et al. (2019) was unspeeded). To compute simulated ERPs, the activity of all lexical units was summed on each time step.

### EEG processing

For both experiments, data was kindly made available to us by the authors. Preprocessing was done using MNE software in Python (Gramfort et al., 2014) and included the following steps: 1) After importing, data were re-referenced to the average of all electrodes (Flanker experiment) or the average of two external electrodes placed on the mastoids (Sentence reading experiment). 2) High-pass (0.1 Hz, 0.1 Hz transition band) and low-pass (30 Hz, 7.5 Hz transition band) basic FIR filters were sequentially applied. 3) An Independent Component Analysis (ICA, Vigario et al., 2000) was run in order to identify and remove eye-blink and eye-movement related noise components (Flanker experiment mean = 1.76 ; Sentence reading mean = 3.92 of first 30 components removed per participant). Noise components were identified using a combination of manual and algorithm-based steps. Initially, for each dataset, we manually identified clear examples of an eye-blink and an eye-movement related component. These manually identified components were then used as templates in mne.preprocessing.corrmap for pattern matching across subjects (correlation threshold 0.8; Flanker median = 0.991 and 0.952 for eye-blink and eye-movement noise components; Sentence reading median = 0.941 and 0.963). Following automated pattern matching, we conducted a manual review of all ICA sources for each subject to ensure accuracy and consistency in the exclusion of components. In total, 1.76 (Flanker experiment) and 3.92 (Sentence Reading) of the first 30 components were removed per participant. There was thus a notable difference in the number of components excluded between the two tasks. We hypothesize that the increased demand and potential differences in eye movement patterns in sentence reading, as opposed to simple fixation in the flanker task, led to the identification and exclusion of a greater number of noise components. 4) epochs were extracted from –200 ms to 900 ms from stimulus onset. 5) trials were normalized by their 200 ms pre-stimulus baseline.

ERPs were computed as in the original papers; only trials for which target words were correctly identified were included, and the N400 interval was defined as in the original studies.

## Results

### Flanker lexical decision task: behavior

**Figure 2:**
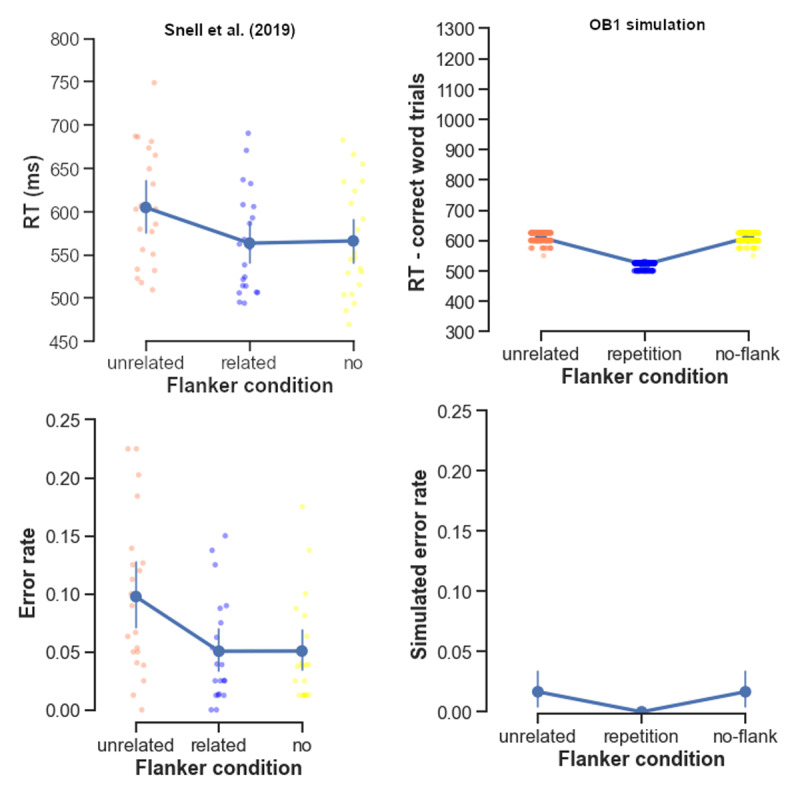
**Average response times (top panels) and error rates (bottom panels) in flanker task.** Left panels: human performance data from Snell et al., (2019) replotted; right panels: OB1 simulation results. Dots indicate individual participants’ average measure, solid lines indicate the group average. Error bars represent the 95 confidence interval.

We first simulated the behavioral results of Snell et al. (2019) obtained with the Flanker paradigm. Their participants made more errors in lexical decision and were slower when unrelated words flanked the target words. Whether the target was repeated as flankers or presented alone made no difference. As shown in Figure 2, OB1 replicated the benefit of related (i.e., repeated) flankers over unrelated flankers, but wrongly predicted that related flankers would outperform the no-flanker condition. We return to this failure in the discussion.

### Flanker lexical decision task: ERP results

**Figure 3:**
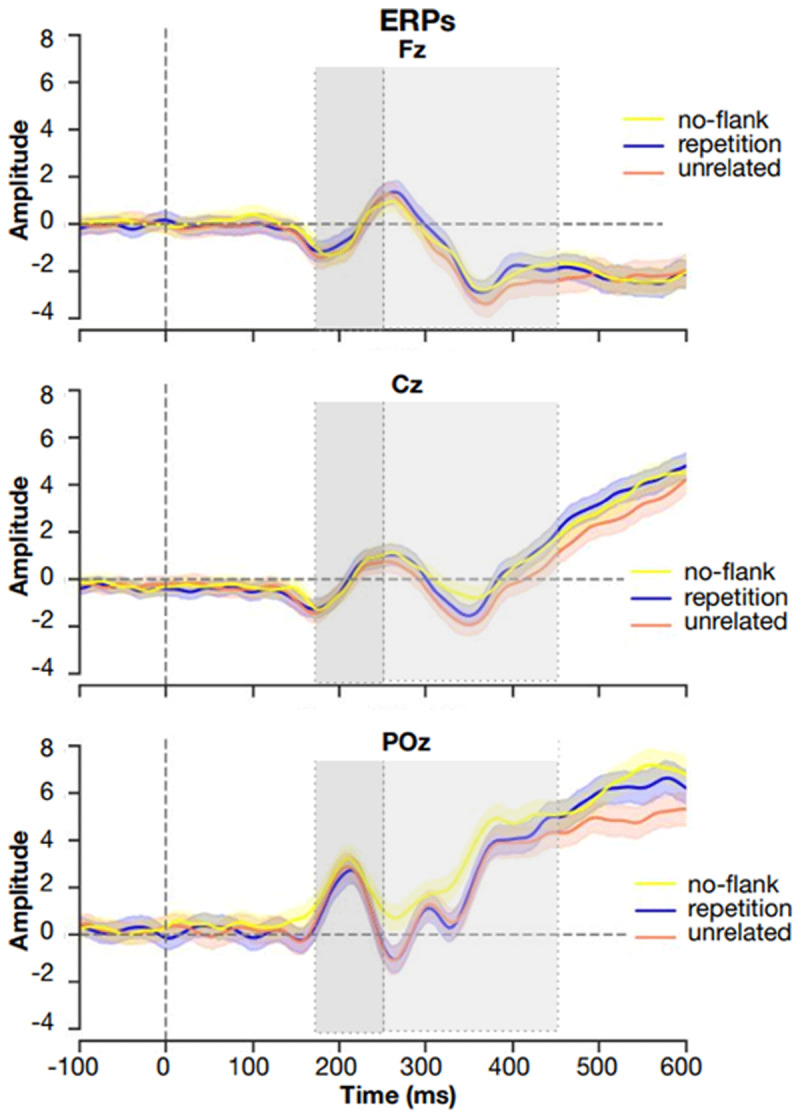
**ERPs for each condition at electrode sites Fz, Cz and Poz.** Figure replotted using the data of Snell et al., (2019).

**Figure 4:**
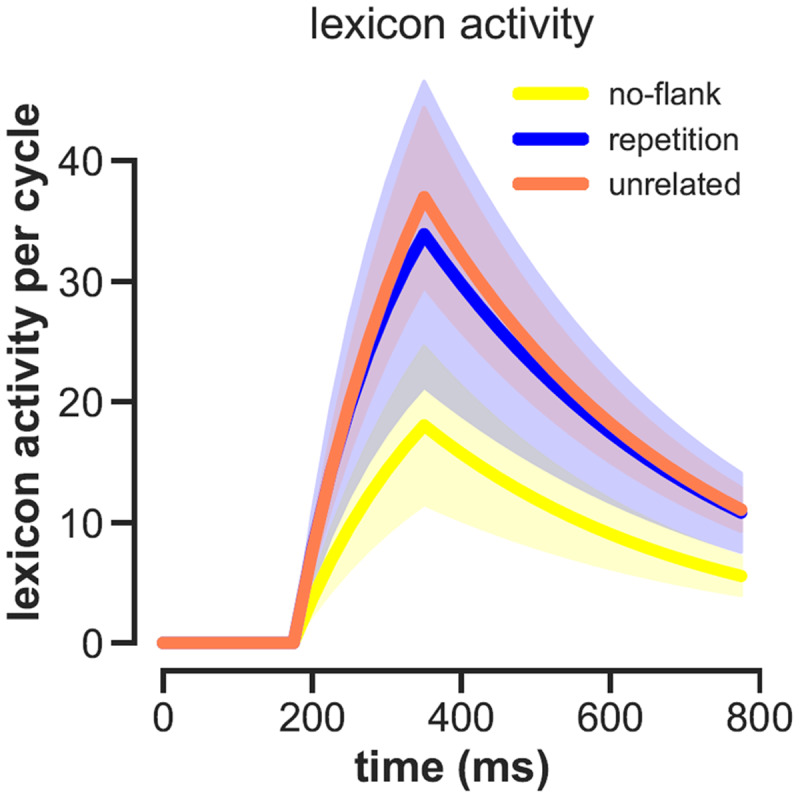
OB1 lexical activity generated by stimuli from the different conditions.

For the human ERPs, differences between the unrelated and repetition flanker conditions became significant around 200 ms and continued beyond 500 ms (see Figure 3). Deflections in the N400 window were more negative in the unrelated flanker condition, and least negative in the no-flanker condition. As Figure 4 shows, OB1 reproduces these differences: least lexical activation is generated in the no-flankers condition, then in the repeated, and finally the unrelated condition.

**Figure 5:**
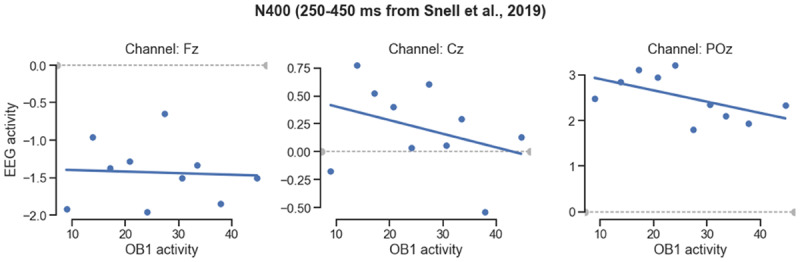
**Correlation between OB1 lexical activity and the N400 from Snell et al. (2019).** Data from Snell et al. taken at 400 ms, with stimuli binned on the basis of the generated lexical activity in OB1 for the same stimuli.

We then tested whether OB1 lexical activity can predict the size of the N400, and thus may provide a good model for human EEG. To investigate this, OB1’s ‘virtual EEG activity’ was computed on every trial, and then binned in ten bins on the basis of OB1’s activity at 400 ms post-stimulus. Next, we obtained the evoked EEG activity from the human participants for the stimuli in each bin, ignoring which condition they were from. Evoked EEG activity for the stimuli in each of these bins was obtained and averaged 250–450 ms (the N400 window used by the original authors). Because participants were presented with different stimuli (target words were presented in each condition, but only in one condition per participant), each stimulus was seen by maximally six participants. A negative relation was expected as a larger N400 translates into a more negative ERP. As shown in Figure 5, there were indeed trends towards a larger N400 at the Cz and Poz electrodes for stimuli where OB1 generated more lexical activity. Computed over items, these correlations was at trend level for electrode POz (r = –.065, p = .08), the electrode where Snell et al. (2019) reported their largest N400 effects.

### Sentence task: behavioral data

**Figure 6:**
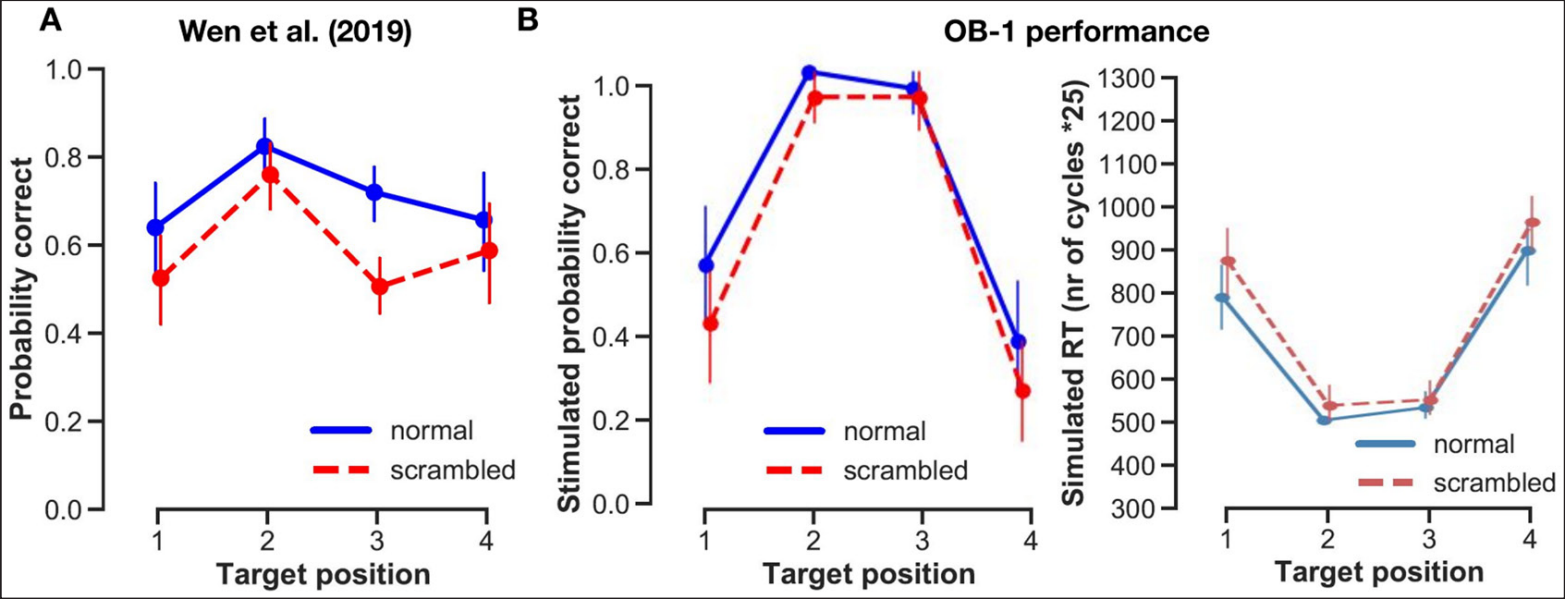
**Behavioral data from Wen et al. (2019) sentence task and OB1 simulations. A)** Human accuracy data from Wen et al., (2019) replotted. **B)** OB1 simulations: percentage correct and **C)** time that it took the model to recognize the words (simulated RT). Error bars represent the 95 confidence interval.

Next, we investigated the sentence task. Participants in the experiment of Wen et al. (2019) were better at reporting words from the normal sentences than from the scrambled ones (see Figure 6). This represents the so-called sentence superiority effect that was the target of the experiment. Moreover, words were recognized better at the second and third positions than at the flanks, presumably because participants most often fixated in the middle. OB1 was able to simulate the effect of position on word recognition, although the effect in the model was much larger than it was in the data. Using the newly introduced syntactic predictions, OB1 could also simulate the sentence superiority effect: words were recognized more readily in intact sentences than in scrambled ones. This effect was evident for all positions but was very weak for the third. The third word was often recognized first in the simulations, and then helped recognize words on the other positions because it allowed their part of speech to be predicted (.e.g., in the sentence “ces loups vont voir” [those wolves will see], vont is a verb which led OB1 to expect a noun before it).

OB1 also generated response times that mirrored the accuracy data. Response times were not reported by Wen et al. (2019), but are given in the Figure 6 as a prediction.

### Sentence task: ERP results

**Figure 7:**
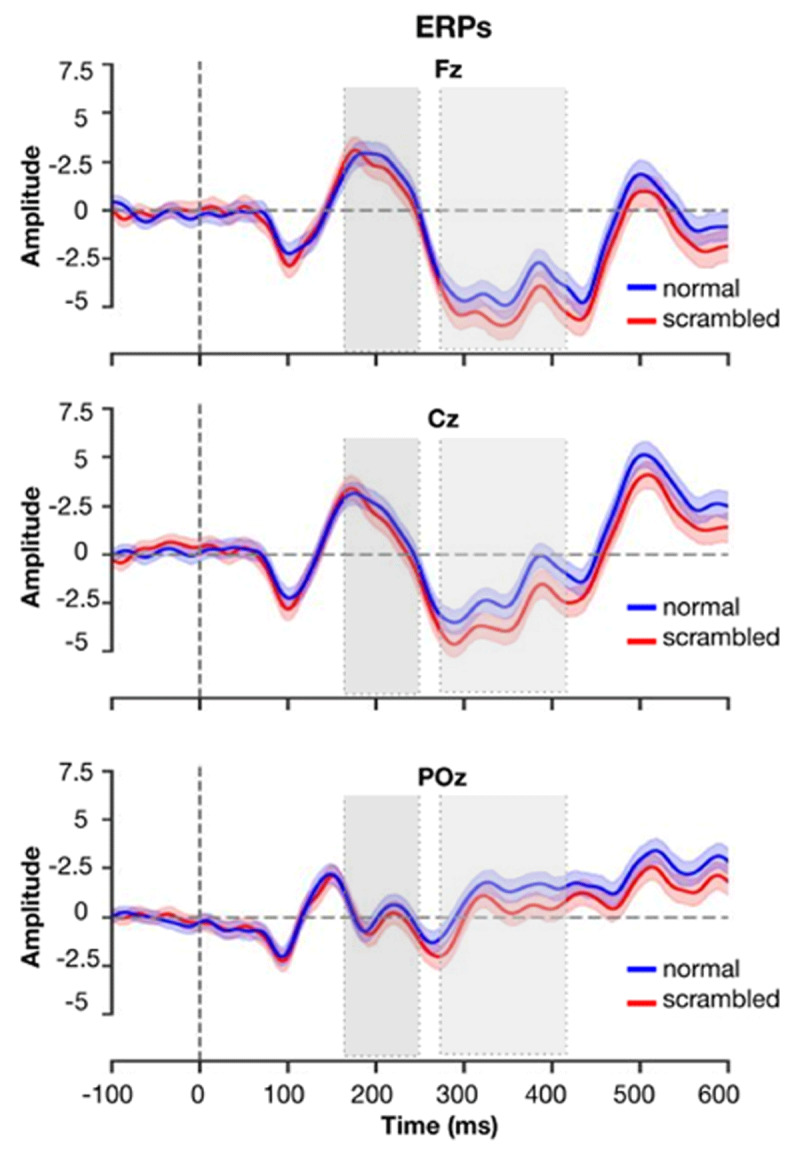
**ERPs generated by normal and scrambled sentences at electrodes Fz, Cz and Pz.** Figure replotted from data of Wen et al., (2019). Light gray bars show the N400 interval used by Wen et al., dark gray bars their N250 interval.

Figure 7 shows the replotted ERPs for three central electrodes (Fz, Cz and POz), with the N400 window (defined as in Wen, Snell, & Grainger, 2019) indicated by the light gray square. Wen et al. focused on the Cz electrode at the center midline, but the effect was widely distributed.

**Figure 8:**
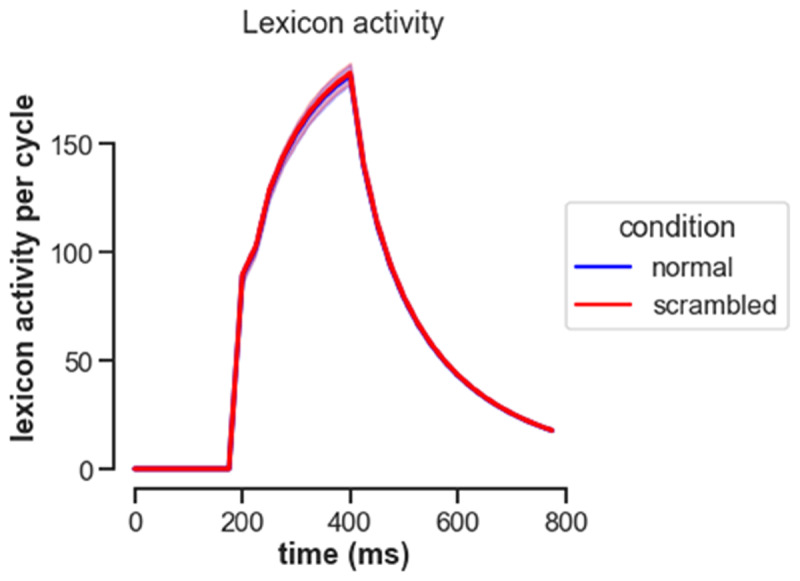
**Simulated N400 for normal and scrambled sentences.** The simulated N400 was generated by summing activity of all OB! lexical units while presenting the stimuli of Wen et al. (2019).

OB1 produced a somewhat larger simulated N400 for scrambled sentences than for intact ones, but the effect was very small (see Figure 8 – note that more lexical activity was taken to simulate a larger N400, so a larger value in Figure 8 would model lower amplitude in Figure 7).

**Figure 9:**
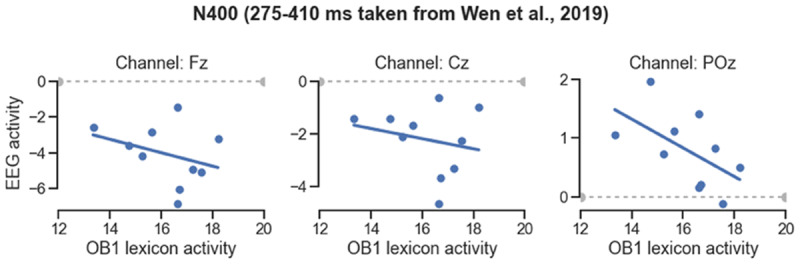
**Correlation between OB1 lexical activity and N400 size in the sentence task.** OB1-generated lexical activity was measured at 400 ms, N400 was computed from the EEG data from the Fz, Cz and Pz electrodes of Wen et al. (2019), using their interval. Stimuli were binned on the basis of OB1’s generated lexical activity.

All word sequences were counterbalanced across conditions and thus presented to a participant in only one of the two (normal and scrambled). We therefore ignored condition so as to be able to generate an ERP per bin for each participant. We computed an N400 per bin using the interval of the authors (275–410 ms; N400), and evaluated whether there was a correlation between these N400s and OB1’s virtual ERPs. As Figure 9 shows, there was a negative relationship between OB1 lexicon activity and EEG activity in all three channels. Computed with individual items as the unit, this correlation was significant for electrode Fz (r = –.154; p = .029), and at trend level for electrode Cz (r = –.133, p = .059).

To test whether this effect could be driven by the number of bigrams or the number of letters in the stimuli, we performed additional analyses in which trials were binned on these two quantities, but this did not lead to correlations with the human N400.

## Discussion

In the current study, we evaluated the ability of OB1-reader to not only capture behavioral phenomena in two experimental tasks, but also to predict electrophysiological responses recorded during those tasks. As far as we know, this is the first attempt to do so in the reading literature. Moreover, several models exist of electrophysiological responses generated during reading, most focusing on the N400 (Cheyette & Plaut, 2017; Laszlo & Plaut, 2012; Nour Eddine, Brothers, & Kuperberg, 2022; Rabovsky, Hansen, & McClelland, 2018; Rabovsky & McRae, 2014). However, the latter tend to not model behavioral responses.

The simulations show a number of successes: OB1 was able to simulate behavioral results reasonably well for both the flanker task, where it captured the detrimental effect of the unrelated flankers, and the sentence task, where it captured both the sentence superiority effect and aspects of the effect of position (see below for deviations between model and data in position effects). With EEG, it successfully generated larger N400s for unrelated than related flankers, and for related flankers than a condition without flankers (Snell et al., 2019). It also generated larger N400s for scrambled than for intact sentences, although this effect was an order of magnitude smaller than the effect seen in human ERPs. These successes were reached without changing OB1’s default parameter settings.

Moreover, for both experiments lexical activity generated within OB1 by particular stimuli could be used to predict the size of the N400 for those stimuli to some extent. Again, this was reached without any parameter fitting, and since the experimental trials could only be binned after the OB1 simulations were run, this constitutes a true, blind test of the model. The success of the simulations suggests that, first, OB1 is able to generate lexical activity resembling that generated in the brain by stimuli. It does this on the basis of orthographic rather than semantic features (as the latter have not been implemented in OB1). Indeed, orthographic features such as orthographic neighborhood size (Holcomb, Grainger, & O’rourke, 2002; Laszlo & Federmeier, 2009). Second, it suggests that activation of the lexicon is a factor in generating the N400. It may be that lexical activation itself contributes to generating the N400. Alternatively, it may be that lexical entries activate semantic features, and that this second step is reflected in the N400 (Bentin, Kutas, & Hillyard, 1995; Cheyette & Plaut, 2017).

### Comparison with other models of the N400

In a recent review, Nour Eddine and colleagues (2022) divided computational models of the N400 into two classes, so-called word-level and sentence-level models. Word-level models (Cheyette & Plaut, 2017; Laszlo & Plaut, 2012; Rabovsky & McRae, 2014) focus on simulating paradigms in which a single word is presented, or in which words are presented in succession as in priming studies. They can simulate reductions in the N400 to words that are repeated, are preceded by related primes, have smaller neighborhood size, are semantically poorer, or have higher frequency. Sentence-level models, on the other hand, simulate N400 effects seen when participants read or hear whole sentences (e.g., Rabovsky & McRae, 2014). These models, can, for example, simulate a smaller N400 to words that fit the preceding context (she put on her hat) than to words that are surprising given the preceding context (she put on her dog). Both categories of models can thus successfully simulate some findings. Nour Eddine et al. (2022) also point out some deficiencies. First, while a few models can generate both the rise and fall of the N400 in time (e.g., Laszlo & Plaut, 2012), most models only generate a monotonously rising function. Second, the model output that is compared to the human N400 is in many models biologically implausible and does not have an obvious function – its only function seems to be to model the N400. Third, each model only simulates a limited set of findings. Fourth, none can model behavioral responses. Nour Eddine (2022) themselves present a model, based on predictive coding theory, that remedies many of these deficiencies. It models the N400 as prediction error, which rises and falls over time in an, according to Nour Eddine et al. (2022), biologically plausible way. Since it incorporates both orthographic and semantic prediction error, it can model both word-level and sentence-level findings. However, it also suffers from the fourth deficiency, not being able to model behavior.

OB1 is in many ways the mirror image of the models discussed by Nour Eddine et al. (2022). The number of findings it can simulate from the N400 literature is currently limited, but it can simulate reading behavior since that is its original goal. Moreover, it generates its in-silico ERP from a plausible output, namely the sum of all lexical activity. Indeed, a leading theory suggests that EEG results from the sum of simultaneous dendritic activity within layers of pyramidal neurons (Murakami & Okada, 2006). Summed lexical activity would thus be a natural source of an ERP component. However, it is possible that transformations of lexical activation, such as a weighted sum with higher weight for highly activated lexical representations, might provide a better fit.

### Instructive failures

Nevertheless, within the simulations, OB1 exhibited a number of instructive failures.

Behaviorally, in the flankers task, OB1 incorrectly predicted better performance for the repeated flankers condition (ROCK ROCK ROCK) than for target words presented alone (ROCK), even though human recognition in these two conditions was equivalent. OB1 assumes that if letters and bigrams are repeated in the input, their activation sums. This may be a faulty assumption – in may be that centrally presented letters and bigrams already generate close to maximal activation of their representations – in which case ‘ROCK ROCK ROCK’ would generate a similar activation pattern as ‘ROCK’.

In the sentence task, OB1 produced stronger position effects than were seen in the data, with high recognition of words in the middle positions and rather low recognition (around 20% instead of 40% as in the data) at the edges. Partly, this may be due to that the current implementation of syntactic constraints is simplistic. It relies only on part-of-speech transitions between adjacent words, while syntactic analysis is generally thought to rely on deep structure. Moreover, longer-range constraints are obviously also important in setting up syntactic expectations.

Another factor is that OB1 is programmed to hold its attention straight at the center, while human observers may be able to covertly move their attention within the 200 ms presentation time. However, modeling such covert attention shifts would be hard to constrain with data as the shifts are, by definition, unobservable. Moreover, also without them OB1 captured the qualitative pattern of better recognition for the center of the sentences than for the edges.

With regard to the N400, figures 4 and 8 feature plausible rise and fall times for the N400. However, in the current simulations these resulted from the presentation times used in the experiments – the end of the 200 ms presentations resulted in a drop of activity that would otherwise not be there. Plausible mechanisms for a return of activity to baseline still have to be implemented in OB1.

Moreover, the N400 is not the only ERP component that has been studied in the word recognition and reading literatures. Next to the N400, we also made an attempt to predict the N250 component from OB1 outputs. However, our simulations were unsuccessful – none of the output measures we generated within OB1 turned out to be a good predictor for the N250. This could mean that there are fundamental differences between orthographic processes as modelled in OB1 and how the brain processes orthography. In particular, it could be that orthographic codes encode the frequency with which bigrams occur in the language in ways that are not captured by OB1 (Snell, in press). It may also be a more benign failure to find a fitting measure of processing within the OB1 model.

## Conclusions

Here, we showed that OB1 reader can simultaneously predict behavioral and N400 ERP data in two paradigms in the literature on word recognition and reading. This was done without parameter fitting or changes to the OB1 model as conceived for other simulations (Meeter et al., 2020; Snell et al., 2018). While this is a promising result, only a limited number of findings from the very large N400 literature were simulated. Future work will have to include a broader range of processes to present a fuller picture of the N400 and other reading-reated ERP components. Testing models’ ability to account for brain activity in addition to behavior offers a valuable way to adjudicate among theories.

## Data Accessibility Statement

Data and code to reproduce the analyses in this article are available at: https://osf.io/xkwz9/.
